# Knowledge, attitudes, and practices of cardiac rehabilitation among physiotherapists in Morocco

**DOI:** 10.21542/gcsp.2024.23

**Published:** 2024-04-20

**Authors:** Kaoutar Kabbadj, Safae El Haddaoui, Nora Taiek, Abdelkader Jalil El Hangouche

**Affiliations:** 1 Department of Physiology, Faculty of Medicine and Pharmacy of Tangier, Abdelmalek Essaadi University, Tangier, Morocco

## Abstract

Cardiovascular disease is a growing challenge worldwide, and Morocco is no exception. Despite its growing popularity, cardiac rehabilitation is still largely underutilized. In order to improve the provision of cardiac rehabilitation, a study of the knowledge, attitudes, and practices of physiotherapists in Morocco, key players in cardiac rehabilitation, is described. This assessment highlights existing skill gaps and enables the necessary corrective measures to be identified. This cross-sectional study included Moroccan physiotherapists working in different institutions (public and private) in different regions of Morocco. A total of 145 valid questionnaires were collected (33% response rate). The results showed that 72.4% of respondents had a medium level of knowledge of cardiac rehabilitation, 93.8% had a positive attitude, and 73% of physiotherapists said they sometimes practiced cardiac rehabilitation. Subgroup analysis showed that the lower the level of education, the more negative the knowledge and practice of rehabilitation. The results also showed that physiotherapists working in education sector and private clinics had better practice than those working in hospitals.

## Introduction

In the past three decades, there has been a significant surge in the worldwide prevalence of diseases. The main drivers of morbidity and illness have transitioned from communicable diseases, maternal and perinatal conditions to non-communicable diseases (NCDs), with a particular emphasis on cardiovascular disease (CVD)^1^, which encompasses stroke, coronary heart disease (CHD), myocardial infarction (MI) and rheumatic heart disease^2^.

CVD has become a major global public health concern, given its widespread occurrence and substantial impact on morbidity and mortality. In 2016, the World Health Organization (WHO) reported that CVD accounted for 17.9 million deaths, 31% of all deaths^1^. Projections indicate that cardiovascular disease will persist as the leading cause of death worldwide in the forthcoming years^3,4^, with an estimated 23 million deaths attributed to these conditions by 2030^5^.

The seriousness of CVD is due to the wide spectrum of manifestations, ranging from coronary heart disease to heart failure, stroke, cardiac rhythm disorders and peripheral vascular disease, among others. These conditions can lead to significant deterioration in cardiac function, physical limitations, reduced quality of life and an increased risk of serious, even fatal, complications.

Cardiovascular disease is also a major health problem in Morocco. It represents one of the main causes of death and disability in the country. In 2015, NCDs accounted for 80% of fatalities, with cardiovascular disease emerging as the primary contributor to death (38%), followed by cancer (18%), and chronic respiratory disorders (6%)^1^. Between 2007 and 2017, the leading cause of death was ischemic heart disease (22%), followed by stroke (15%). Associated risk factors, such as high blood pressure, smoking, obesity and diabetes, are also widespread and contribute to the growing burden of CVD in the country. For example, diabetes-related heart disease mortality rose from ninth to fourth place as a cause of death (an increase of 35.4%) between 2007 and 2017, and hypertension-related heart disease mortality rose from tenth to sixth place as a cause of death (an increase of 27.6%) over the same period^1^.

Although CVD is incurable, pharmacological and non-pharmacological therapies can minimize exacerbations and hospitalizations^6^. Cardiac rehabilitation (CR) is a mainstay of non-pharmacological therapeutic techniques for people with CVD^7^.

CR is considered a cost-effective approach for secondary prevention, aiming to stabilize, slow down, or potentially reverse the course of cardiovascular disease^8^. It is defined by the World Health Organization (WHO) as the “sum of activities required to influence favourably the underlying cause of the disease, as well as to provide the best possible physical, mental and social conditions, so that the patients may, by their own efforts, preserve or resume when lost, as normal a place as possible in the community”^5^.

CR programs provide a comprehensive intervention that includes physical training, physical activity promotion, health education, cardiovascular risk management and psychological support, all tailored to address the unique requirements of individuals with heart disease. CR is associated with several validated benefits consisting of healthy lifestyle promotion, risk factors prevention, and health-related quality of life enhancement^9,10^, as well as reducing 5-year cardiovascular mortality from 25% to 46%^11,12^ and hospital readmissions for cardiovascular events by 28%^13,14^. This is a Class 1, Level A recommendation in clinical practice guidelines for cardiac patients^10,15,16^.

Despite the benefits and clear recommendations on the efficacy and importance of cardiac rehabilitation, it remains under-utilized worldwide^7,17,18^. Only a small proportion of patients with cardiovascular disease benefit from this therapy, partly due to a lack of qualified personnel and partly due to a lack of cardiac rehabilitation centers^19^. The situation in Morocco is not much better. To the best of our knowledge, Morocco has only two cardiac rehabilitation centers for cardiovascular disease patients in the public health system. To improve the implementation of CR programs in Morocco, it is necessary to assess the knowledge, attitudes and practices of physiotherapists towards these programs, but also to understand the obstacles they encounter in the practice of cardiac rehabilitation in Morocco.

Physiotherapists play a decisive role in the implementation, follow-up and success of any cardiovascular rehabilitation program. They have a key role to play in improving patients’ cardiovascular health, strengthening their physical condition, managing cardiovascular risk factors, preventing long-term complications and promoting a better quality of life. Indeed, physiotherapists need to know how to conceive personalized exercise programs for each patient, based on initial assessment; they are required to carefully monitor patients during exercise sessions to detect any signs of cardiac distress or potential complications; and they are required to encourage patients to adopt a healthy lifestyle by giving them advice on physical activity, balanced diet, smoking cessation, stress management, etc. To achieve this, physiotherapists must have knowledge and skills in the field of cardiac rehabilitation.

The purpose of the present study is to comprehensively evaluate the knowledge, attitudes and practices (KAP) of Moroccan physiotherapists in the context of CR. This research is also an attempt to shed light on the roles played by these professionals within CR domain and to identify the obstacles they encounter in their daily practice. In the course of this study, we intend to pinpoint any prevalent gaps in their knowledge, practice and training, thus determining the strengths and weaknesses in the physiotherapists’ understanding and implementation of CR. The conduct of this assessment marks the initial step in discerning the necessary educational initiatives, specific training and awareness programs required to enhance the state of CR within the Moroccan healthcare system. This, in turn, ensures that physiotherapists are equipped with the essential knowledge and tools in the field of CR to provide the highest quality of care to their patients.

## Materials and Methods

### Study design and settings

This is a cross-sectional study targeting physiotherapists working in hospitals, private clinics and education sector in different regions of Morocco. Data were collected between July 12, and September 30, 2023 using a questionnaire distributed electronically. Inclusion criteria were: practising physiotherapists. Exclusion criteria were: physiotherapy students and other healthcare professionals.

This study was conducted in compliance with the principles of the Declaration of Helsinki. Participation in this study was entirely voluntary and every participant was informed that they could refuse or withdraw from the study at any moment.

### Study participants and sampling

A convenient sampling method was used to recruit study participants^20^. The target population consisted of physiotherapists who had recently attended a national conference on physiotherapy. Conference attendees were from different institutions (public and private) and different regions of Morocco. Among the list of conference participants, 433 email addresses were identified. The questionnaire was distributed *via* a web-based email service, yielding 213 responses. Subsequently, 68 responses were excluded due to incomplete answers. Ultimately, the participant sample for the study consisted of 145 physiotherapists.

### Questionnaire tool

The KAP questionnaire used in our study was designed on the basis of the literature. It was then validated by a panel of experts in cardiac rehabilitation, including both experienced physiotherapists and cardiologists. Their insights critically influenced the tool’s development, ensuring it was thoroughly adapted to reflect the nuances of cardiac rehabilitation practices within the Moroccan healthcare context. The feedback from these experts prompted significant refinements to the questionnaire.

Prior to its finalization, the questionnaire underwent a pilot test with a select group of physiotherapists not involved in the main study. This critical step helped identify and rectify any ambiguous questions or terms, thereby refining the tool further to ensure consistency in interpretation across all respondents.

The final version of the questionnaire was structured into six distinct sections. Originally adopting the WHO KAP framework that includes sections on knowledge, attitudes, and practices, we expanded this model to include three additional sections. These were designed to gather comprehensive socio-demographic data, assess the perceived role of physiotherapists in cardiac rehabilitation, and identify specific obstacles encountered by physiotherapists in this field. This expansion aimed to not only capture a snapshot of the current state of cardiac rehabilitation knowledge and application but also to delve deeper into the systemic and individual factors that may affect the implementation of effective rehabilitation practices:

• Part 1 covered the socio-demographic data of the respondents : It is worth mentioning that, concerning the diplomas evaluated in our study, the selection of modalities for this variable was influenced by the different types of diplomas inherent in the Moroccan education system. That said, in Morocco, physiotherapy training is organized around two main educational courses. On the one hand, it can be provided within professional training schools, where students obtain the “Specialized Technician Diploma in Physiotherapy” at the end of their course. This program focuses on practical skills in the field of physical therapy. On the other hand, training in physiotherapy can also follow the university curriculum (Bachelor’s, Master’s, Doctorate). In this case, students can earn a bachelor’s degree in physical therapy after the first cycle of study, and have the opportunity to continue on to a master’s degree, or even a doctorate, if they wish to deepen their academic knowledge, practical skills and research. This duality of training can influence the depth of theoretical knowledge, attitudes and practice of physiotherapists in cardiac rehabilitation.
• Part 2 focused on physiotherapists’ knowledge of CR
• Part 3 explored physiotherapists’ attitudes to CR.
• Part 4 assessed physiotherapists’ CR practices.
• Part 5 explored the role played by physiotherapists in cardiac rehabilitation.
• Part 6 explored the obstacles encountered by physiotherapists in the practice of cardiac rehabilitation.

We employed a 5-level Likert scoring method to evaluate the “knowledge and practice” components in our study. For the “knowledge” dimension, responses were graded on a scale ranging from 1 for “poor” to 5 for “excellent”, while the “practice” component utilized a scale ranging from 1 for “never” to 5 for “always”. Conversely, the “attitude”, ”role”, and “obstacles” components were appraised using a two-level Likert scale, with scores ranging from 0 for “disagree” to 1 for ”strongly agree”. Attitudes were categorized as negative if the score was 2 or below, and positive if above 2. To ensure the reliability of our measurement tool, we conducted an internal consistency analysis, calculating Cronbach’s alpha, yielding a robust result of 0.770. This high alpha value signifies strong consistency among the questionnaire items, reinforcing the reliability of our methodology. Consequently, this enhances the validity of our approach, underscoring the robustness of the data collected. This meticulous evaluation allows for a comprehensive examination of the knowledge, attitudes, practices, and roles of physiotherapists in Morocco.

Each participant was informed of the purpose of the study, and the questionnaire began with a question asking participants if they were interested and consented to take part. The time allowed for completing the questionnaire was approximately three to five minutes.

### Statistical methods

The Statistical Package for the Social Sciences version 26.0 (SPSS, Chicago, IL, USA) was used for statistical analyses. Data distributions are analyzed using the Kolmogorov–Smirnov test. Continuous variables (knowledge and practice) are described as mean ± standard deviation or median (IQR) as appropriate. Two-sample t-tests or Mann–Whitney U-tests are used to compare data between genders, and one-way ANOVA tests or Kruskal-Wallis H-tests followed by the post-hoc Mann–Whitney U-test to compare data between the subgroups of each variable: work experience, geographic location, work setting and type of diploma, depending on whether or not the quantitative data conform to a normal distribution.

Categorical variables (attitude) are presented as frequencies and percentages and are analyzed using the chi-square test or Fisher’s exact test as appropriate. For all analyses, 95% confidence intervals (CIs) are reported, and a two-tailed *p* value < 0.05 was considered statistically significant. To assess the reliability of the questionnaire, Cronbach’s alpha was employed to measure internal consistency.

## Results

A total of 145 physiotherapists, (72% women, 28% men) completed the online survey between July 12 and September 30, 2023. The mean age of the participants was 31 (27–34) years. Of the respondents, 68.2% had less than 10 years’ professional experience. Respondents represented different regions of the Kingdom, as follows: 37.2% from the Northern region, 33.8% from the Central region and 29% from the Southern region of Morocco. Table 1 summarizes the sociodemographic characteristics of the participants.

**Table 1:** Basic characteristics of participants (*n* = 145).

**Characteristics**	**n(%)**	**95% Cls**	**Median (IQR)**	**95% Cls**
Age			31 (27–34)	30–32
Gender				
- Female	104 (71.7)	64.1 –78.6		
- Male	41 (28.3)	21.4 –35.9		
Work experience (years)				
- <5	55 (37.9)	30.3–46.2		
- 5–10	44 (30.3)	23.4–37.9		
- 10–15	28 (19.3)	13.1–26.2		
- >15	18 (12.4)	7.6–17.9		
Geographic Location				
- Northern region	54 (37.2)	29–45.5		
- Central region	49 (33.8)	26.9–41.4		
- Southern region	42 (29)	22.1–37.2		
Work setting				
- Hospital settings	64 (44.1)	36.6–52.4		
- Private clinic	65 (44.8)	36.6–53.1		
- Education sector	16 (11)	6.2–16.6		
Type of diploma				
- Specialized technician diploma in Physiotherapy	59 (40.7)	33.1–48.3		
- Bachelor’s degree in Physiotherapy	80 (55.2)	46.9–63.4		
- Master’s degree or higher in Physiotherapy	6 (3.4)	1.4–7.6		

**Notes.**

95% CIs: 95% confidence intervals for proportions and median.

IQR Interquartile rang.

### Knowledge of CR

**Figure 1.**
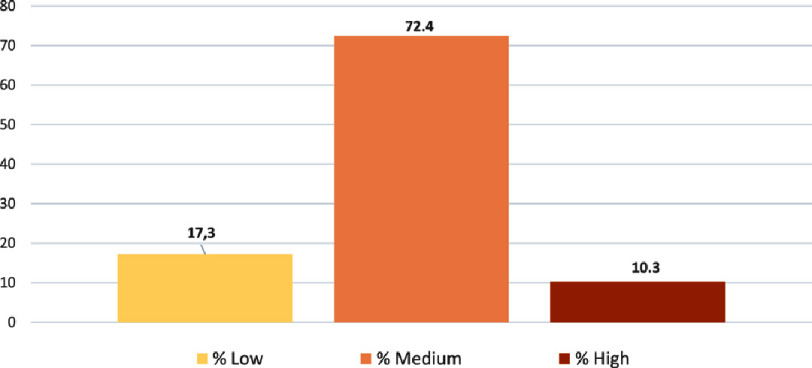
Overall level of knowledge of cardiac rehabilitation among Moroccan physiotherapists.

The results revealed that almost half of participants 47.6% ( *n* = 69) reported having a medium level of knowledge of CR, and 31% ( *n* = 45) reported having a low level of knowledge of CR. In terms of program content, 66.3% of participants ( *n* = 96) reported a low to moderate level of knowledge of the multidisciplinary components of CR, and 33.7% ( *n* = 49) reported a good to excellent level of knowledge of the multidisciplinary components of CR. The majority of respondents 58.6% reported a low to moderate level of knowledge of the main objectives of cardiac rehabilitation, and 29.7% a good level. The results of the knowledge questionnaire were summed up and expressed in the form of an overall score, and which is shown in the Figure 1.

### Attitude towards CR

**Figure 2.**
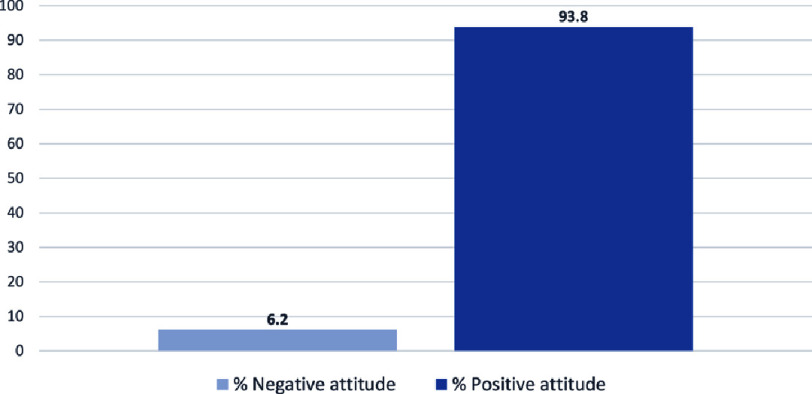
Attitude of physiotherapists towards cardiac rehabilitation.

The results show that more than 90% of physiotherapists have a positive attitude towards cardiac rehabilitation, recognizing its importance in the recovery process of cardiac patients, in improving their quality of life, and in changing cardiac patients’ behavior towards cardiovascular risk factors. However, most of our survey respondents (87%) stated that cardiac rehabilitation was not sufficiently developed in Morocco. The scores of the attitude questionnaire were summed up and expressed as positive or negative attitude proportions, and are presented in Figure 2.

### CR practice among physiotherapists

Our results showed that over a quarter of respondents (26.9%) had never treated patients with cardiovascular disease in the past month, 34.5% reported rarely treating cardiac patients in the past month, and only 12.4% of participants reported often treating patients with cardiovascular disease in the past month.

Our results also showed that 25.5% of physiotherapists surveyed always recommend cardiac rehabilitation to their patients, and 34.5% of respondents recommend adapted physical exercise to their patients.

In relation to participation in cardiovascular rehabilitation training courses, 35.7% of the surveyed individuals reported never attending such courses, while only 3.6% stated that they often attended.

**Figure 3.**
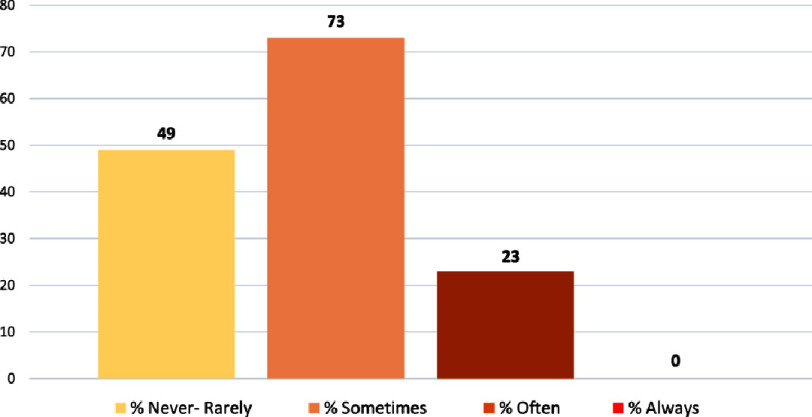
The extent of cardiac rehabilitation practice and implementation among Moroccan physiotherapists.

An overall score was calculated by summing the scores of the questionnaires relating to cardiac rehabilitation practices. This score is used to describe the extent to which physiotherapists in Morocco implement cardiac rehabilitation. this finding is illustrated in Figure 3.

### Barriers to the practice of CR in Morocco

**Figure 4.**
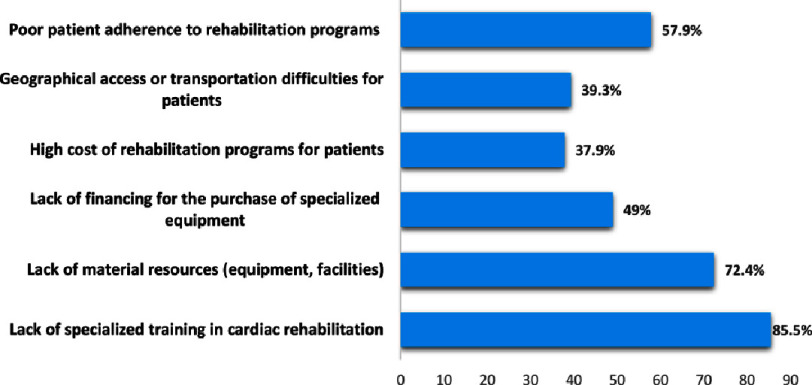
Barriers to the practice of cardiac rehabilitation in Morocco.

The study also identified several barriers to the practice of cardiac rehabilitation by physiotherapists in Morocco, including lack of specialized training in cardiac rehabilitation (85.5%), lack of material resources (equipment, facilities) (72.4%) and poor patient adherence to rehabilitation programs (57.9%), more details can be found in Figure 4.

### The role of the physiotherapist in CR

Our results underlined the essential role of physiotherapists in cardiac rehabilitation, as key players in improving patient fitness, promoting their recovery and preventing complications. Over 95% of respondents agreed that the role of the physiotherapist is to develop exercise programs adapted to cardiac patients, and to assess, monitor and improve their physical condition and functional capacity. Respondents also consider that physiotherapists have other key roles in prevention and awareness, such as providing advice and information on healthy lifestyle habits to prevent cardiovascular disease, as well as educating patients on health behaviors conducive to heart disease prevention. Finally, the results show that the majority of physiotherapists (95.2%) are in favor of acquiring additional skills to optimize their role in cardiac rehabilitation.

The statistical analysis of gender, professional experience, geographical location, work setting, and type of diploma subgroups revealed significant differences in knowledge scores between the different types of diploma (*p* = 0.01). The Mann–Whitney post-hoc test showed a statistically significant difference (*p* < 0.05) between those with a master’s degree or higher and those with a bachelor’s degree. More results are presented in Table 2.

**Table 2:** Comparison of knowledge scores between subgroup.

**Characteristic**	**Median (IQR)**	**p-value**
Gender		**0.025**
- Male	7 (6–9)	
- Female	6 (6–8.75)	
Work experience (years)		0.526
- <5	7 (6–9)	
- 5–10	6 (6–9)	
- 10–15	6 (6–8)	
- ¿15	6.5 (4.5–9)	
Geographic Location		0.728
- Northern region	6 (6–9)	
- Central region	6 (6–9)	
- Southern region	6 (6–8.25)	
Work setting		0.315
- Hospital settings	6 (6–8)	
- Private clinic	7 (6–9)	
- Education sector	6.5 (6–10.25)	
Type of diploma		**0.010**
- Specialized technician diploma in Physiotherapy	7 (6–9)	
- Bachelor’s degree in Physiotherapy	6 (6–8)	
- Master’s degree or higher in Physiotherapy	10.5 (6.75–12.5)	

**Notes.**

Bold values indicate statistical significance at *P* < 0.05.

IQR Interquartile rang.

Regarding attitude scores, no significant difference was observed between groups (*p* > 0.05) results are presented in Table 3.

**Table 3:** Comparison of attitude scores between subgroup.

	Negative attitude	Positive attitude	
**Characteristic**	**n(%)**	**n(%)**	**p-value**
Gender			0.118
- Male	5 (55.6)	36 (26.5)	
- Female	4 (44.4)	100 (73.5)	
Work experience (years)			0,239
- ¡5	2 ( 22.2)	53 (39)	
- 5–10	3 ( 33.3)	41 (30.1)	
- 10–15	1 (11.1)	27 (19.9)	
- ¿15	3 (33.3)	15 (11)	
Geographic Location			0.347
- Northern region	3 (33.3)	51 (37.5)	
- Central region	5 (55.6)	44 (32.4)	
- Southern region	1 (11.1)	41 (30.1)	
Work setting			0.142
- Hospital settings	7 (77.8)	57 (41.9)	
- Private clinic	2 (22.2)	63 (46.3)	
- Education sector	0	16 (11.8)	
Type of diploma			0.108
- Specialized technician diploma in Physiotherapy	1 (11.1)	58 (42.6)	
- Bachelor’s degree in Physiotherapy	7 (77.8)	73 (53.7)	
- Master’s degree or higher in Physiotherapy	1 (11.1)	5 (3.7)	

Finally, for the practice component scores, significant differences were observed between different types of diploma, as well as between different work settings (*p* < 0.05). Other results are shown in Table 4.

**Table 4:** Comparison of practice scores between subgroup.

**Characteristic**	**Median (IQR)**	**p-value**
Gender		0.863
- Male	8(4–10)	
- Female	7 (5–10)	
Work experience (years)		0.280
- <5	8 (5–10)	
- 5–10	8 (5–10)	
- 10–15	7(4–10)	
- ¿15	8 (5.75–10)	
Geographic Location		0.730
- Northern region	7 (4–10)	
- Central region	7 (5–10)	
- Southern region	7.5 (5–10)	
Work setting		**0.001**
- Hospital settings	6 (4–8)	
- Private clinic	8 (5.5–10)	
- Education sector	10.5 (6.25–11.75)	
Type of diploma		**0.014**
- Specialized technician diploma in Physiotherapy	8 (6–11)	
- Bachelor’s degree in Physiotherapy	6 (4–8)	
- Master’s degree or higher in Physiotherapy	9.5 (4.75–11.25)	

**Notes.**

Bold values indicate statistical significance at *P* < 0.05.

IQR Interquartile rang.

## Discussion

To the best of our knowledge, this is the first study in Morocco to assess physiotherapists’ knowledge, attitudes and practices towards cardiac rehabilitation, while looking at their role in cardiac rehabilitation and the barriers they face in their practice.

Our results indicate that participants possess an intermediate level of knowledge regarding cardiac rehabilitation. This finding aligns with a similar study conducted by a researcher in Lebanon, who also concluded that physiotherapists lacked sufficient knowledge of cardiac rehabilitation^21^. However, research performed in different other countries indicates that physiotherapists demonstrated a high level of knowledge of cardiac rehabilitation^22,23^. For example, a cross-sectional survey of seven teaching hospitals in Zhejiang province revealed that medical staff including physiotherapists had a good knowledge of cardiac rehabilitation^22^. Similarly, Javed et al. reported that all physiotherapists had a good knowledge of cardiac rehabilitation^23^.

The differences with our results may stem from differences in participation rates in cardiac rehabilitation training activities. Despite the recent increase in such training in China^23,24^, Moroccan physiotherapists perceive a significant obstacle in their cardiac rehabilitation practice, attributing it to a scarcity of training opportunities. Physiotherapists’ knowledge of cardiac rehabilitation is of paramount importance and directly influences the therapeutic process for patients. Sufficient training in cardiac rehabilitation could therefore greatly improve physiotherapists’ knowledge and, consequently, the management of patients with cardiovascular disease.

As described in our results, the majority of physiotherapists (>95%) have a positive attitude towards cardiac rehabilitation and recognize its importance in the recovery process of cardiac patients, in improving patients’ quality of life, and they also believe that CR has the potential to change the behavior of patients with cardiovascular disease. Indeed, the efficacy of cardiac rehabilitation programs has been demonstrated worldwide, with several studies showing that cardiac rehabilitation significantly improves disease-related quality of life, also thanks to the exercise retraining component, CR, improves endothelial function, decreases catecholamine influx, increases peripheral oxygen extraction and increases maximal oxygen uptake^25–27^.

Despite all these benefits and standard international clinical guidelines, cardiac rehabilitation is still considered the ‘Cinderella’ of heart disease treatment. CR programs are available in only 39% of countries, with 68% of high-income countries and 23% of low and middle income countries offering such programs^28^. This is supported by 86.9% of the physiotherapists interviewed in our study who believe that cardiovascular rehabilitation is underdeveloped in Morocco.

This study also provides information on the practice of physiotherapists in cardiac rehabilitation. Our results indicates that 25.5% of Moroccan physiotherapists surveyed systematically encourage their cardiac patients to follow a cardiac rehabilitation program, and 34.5% systematically recommend physical activity in their daily practice. According to several studies, regular physical exercise is recognized as an effective strategy for both cardiovascular disease prevention and rehabilitation. Physical activity has been shown to be beneficial to health in terms of all-cause mortality^29–31^, as well as for various disease risk factors^32–34^. The World Health Organization (WHO) recommends at least 150 min of moderate-intensity PA or 75 min of vigorous-intensity PA per week to achieve these health benefits^35^. In this context, physiotherapists should play a key role in raising awareness and prescribing physical activity and rehabilitation exercises tailored to each patient.

Furthermore, our results show that the frequency with which cardiac patients consult physiotherapists is low (3.4%). This finding aligns with a study carried out in Lebanon^21^ and it may be attributed to a lack of prescription of cardiac rehabilitation by cardiologists or low patient compliance, as reported by our physiotherapists. In this respect, additional research is required to investigate the challenges faced by cardiologists and cardiac patients in prescribing and adhering to cardiac rehabilitation programs.

Regarding the association between the KAP scores and participant characteristics, there were significant differences in the knowledge and practice of physiotherapists regarding the application of cardiac rehabilitation: the lower their level of education, the more negative their knowledge and practice of rehabilitation. This can be explained by the fact that higher education generally allows for more in-depth training and longer immersion in specific areas of physiotherapy, including cardiac rehabilitation. This gives physiotherapists more opportunity to keep abreast of the latest practice and research updates in cardiac rehabilitation. These results are consistent with those of a study conducted in the United States, which clearly demonstrated the benefits of cardiac rehabilitation training^13^. The study revealed a significant increase in the mean score for knowledge of cardiac rehabilitation, from 5.1 before the training activity to 7.0 afterwards (*P* = 0.001).

In addition, our results show that physiotherapists working in education and private clinics sectors have better practice than those working in hospitals, which may be related to the fact that physiotherapists in clinics have often more time to spend with each patient, allowing them to carry out a more comprehensive assessment and follow-up of cardiac rehabilitation. Besides, physiotherapists working in education and clinics have usually more opportunities to attend continuing education courses specializing in cardiac rehabilitation, allowing them to keep up to date with the latest practice and research.

## Implications

In light of the findings from this study, there are substantial implications for clinical practice and healthcare policy in Morocco that warrant careful consideration. The evident knowledge gaps and the varying attitudes and practices of physiotherapists toward cardiac rehabilitation underscore a pressing need for targeted educational interventions. Specifically, enhancing the curriculum in physiotherapy training programs to include more comprehensive, evidence-based content on CR could substantially elevate the competency of future practitioners.

Additionally, the development of continuing education programs could update and expand the existing knowledge base of current practitioners, ensuring that the latest research and best practices in CR are well integrated into daily clinical practice. Furthermore, healthcare policymakers could consider the integration of standardized CR protocols into national health guidelines, which would not only normalize CR practices across the country but also elevate the quality of care provided to patients with cardiovascular diseases.

These steps could significantly contribute to the systematic upliftment of CR services, ultimately improving patient outcomes. Moreover, by addressing these educational and policy-driven changes, Morocco can enhance the implementation and effectiveness of CR programs, thus ensuring that more patients receive the full spectrum of care necessary for optimal recovery from cardiac events. These recommendations not only align with the global movement towards enhancing CR accessibility and quality but also cater specifically to the localized needs and challenges identified through this research, paving the way for a robust healthcare system that supports sustained cardiac health and rehabilitation.

## Limitations of the study

The limitations of this study should be acknowledged. First, the study employed a convenience sampling technique, which may introduce selection bias. Second, the study excluded other healthcare professionals involved in the cardiac rehabilitation process. Third, the data are based on self-reports from physiotherapists, which could introduce a social desirability bias, with participants responding in a way that appears more in line with norms. A future study could consider additional verification methods, such as direct observation of clinical practices, to complement these self-reported data and strengthen the validity of the results.

## Conclusion

The assessment of Moroccan physiotherapists’ Knowledge, Attitudes, and Practices (KAP) in cardiac rehabilitation reveals positive aspects, including favorable attitudes and good practices. Nevertheless, the identified barriers necessitate concerted efforts to overcome obstacles and enhance the effectiveness of cardiac rehabilitation. Physiotherapists need to be supported by adequate resources and strengthened interdisciplinary collaborations to maximize their contribution to cardiovascular health. Further efforts are also needed to strengthen knowledge, promote evidence-based practices and foster a multidisciplinary approach to cardiac rehabilitation. These results will help identify ways to improve and optimize cardiac rehabilitation care delivered by physical therapists, in order to provide patients with quality care and promote their recovery.

## Acknowledgement

The authors are thankful to all the physiotherapists who volunteered to participate in the study.

## Financial support and sponsorship

The authors did not receive support from any organization for the submitted work.

## Conflicts of interest

The authors have no conflicts of interest to declare

## Authors Contribution

**Conceptualization:** Kaoutar Kabbadj, Abdelkader Jalil El Hangouche

**Literature search:** Kaoutar Kabbadj

**Data acquisition:** Kaoutar Kabbadj

**Data and statistical analysis:** Kaoutar Kabbadj, Abdelkader Jalil El Hangouche, Safae El Haddaoui

**Writing- original draft preparation:** Kaoutar Kabbadj, Abdelkader Jalil El Hangouche, Safae El Haddaoui

**Writing- review and editing:** Kaoutar Kabbadj, Abdelkader Jalil El Hangouche, Safae El Haddaoui, Nora Taiek

**Supervision:** Abdelkader Jalil El Hangouche
